# Waterproofing performance of polypropylene – concrete wall of underground silo under combined compressive stress and water pressure

**DOI:** 10.1016/j.heliyon.2022.e12074

**Published:** 2022-12-05

**Authors:** Hao Zhang, Hongkai Wang, Yang Zhou, Zhe Chang

**Affiliations:** a College of Civil Engineering and Architecture, Henan University of Technology, Zhengzhou 450001, PR China; b Henan University of Technology Design and Research Academy, Zhengzhou 450001, PR China

**Keywords:** Silo, Polypropylene, Composite, Bending moment, Adjustment coefficient

## Abstract

Safe, economical and high-quality storage of huge amount of grain for a longer duration under COVID-19 is a challenge and underground storage is a good alternative due to stable temperature, less cooling consumption and better pest control effect. However, the underground silo has very high requirement of waterproof and the performance of underground silo under combined compression and water pressure was rarely studied. In this study, a new composite structure, polypropylene – concrete wall (PPCW) for underground silo was proposed. Total three PPCWs with different size were manufactured to test the waterproofing features under joint effect of compression and hydropower of water. The strains, lateral displacement and cracking conditions of PPCWs were investigated. According to the experimental results, the PP board and concrete presented very good performance of interaction working under compression. The maximum water pressure of the specimens with stud spacing of 250mm increased by about 15.7% compared with that of the specimens with stud spacing of 350mm. The welding and strength of PP board has the greatest influence on the ultimate performance of PPCW. Based on the empirical coefficient method of concrete flat-slab and tested results, a new modified method was proposed to predict the bending moment at mid-span of PPCW by using an adjustment coefficient, *R*_*m*_. Considering this experimental case only, the adopting a *R*_*m*_ = 0.64 could control the relative errors between test and analysis under 15.6%.

## 1 Introduction

According to National Bureau of Statistics [1], the foodstuff sown area was 1176.32 thousand *hectares* including 10,177 thousand *hectares* of grain sown area, and the grain production was 63.276 million *tons* in 2021 in China. Furthermore, due to the outbreak of COVID-19, the stresses on the food systems increased dramatically and strategic grain reserve was increased accordingly from 2020. As reported by FAO [2, 3] and Goswami et al. [4], the grain trade experienced a breaking record of 433 million tonnes. The imported grain (wheat, rice and maize) for at least 25 states (including China, Japan, European Union member) was increased. At the same time, at least 27 states (e.g., India and Russia) limited the export of grain from 2021. For the states which increases the import and those which decrease the export of grain, they need to solve the problem of safe, economical and high-quality storage of such extra huge amount of grain for longer duration under the current situation.

In general, the large bulks of grain usually stored in multi-storied warehouse, flat warehouse, silo, and shallow silo on the ground. However, some researchers [5, 9] recommended to use the underground granary (UG) instead of the on-ground storage. The underground storage takes the advantages of stable temperature of soil layer, thus the cooling consumption can be significantly reduced and drug fumigation for pest control is not need anymore. The use of UGs has a long history [6, 7, 8] and traditional UGs normally requires good geological conditions, e.g., low groundwater levels and no seepage path/cracks for water. Different from the traditional UGs of long time ago, modern UGs should be able to bear a more complex working environment (e.g., undesired underwater level or soil properties) and then applied in more various geological conditions. Some experimental and numerical analyses of UG has been reported before by [9]. Pan et al. [10] developed a new supporting structure of excavation for UGs and Zhang et al. [11] systematically studied heat transfer and ventilation of a UG. Nakashima et al. [12] investigated the feature of floating for a underground silo under high water table. Zhang et al. [13] studied the anti-floating problem of UGs cylindrical grain silo in different soils. Some experimental studied the use of polymers in food package [14].

**Figure 1:**
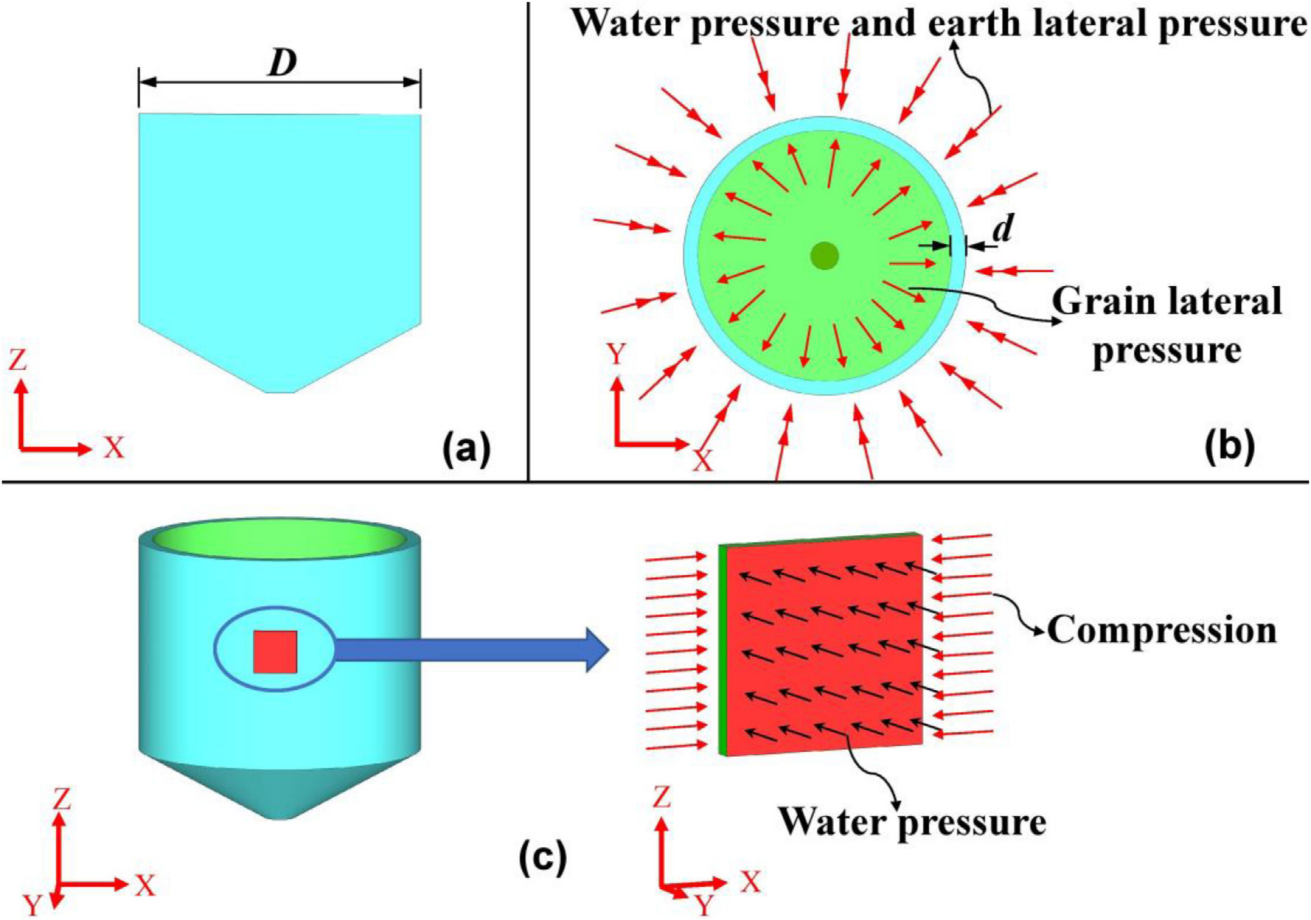
Illustration of underground silo, (a) elevation. (b) Lateral stress, (c) stress on an analysis unit.

However, high water table will yield large static pore pressure (*u*_*s*_) and above engineering/human activities may further induce an additional excess pore pressure (*u*_*e*_). Large pore pressure throws big challenge to waterproofing of the UGs. For example, assume a 30 m deep UG (i.e., underground silo) and a 2.5 m deep water table underground, the soil will yield a *u*_*s*_ of 275 kPa at the bottom of silo. The waterproofing of this silo is a headache to designers and engineers. Up to date, there is rare report on this topic and further study is needed. Another issue of the UGs was ignored previously is lateral stress from outer surrounding soil (*σ*_*h-s*_) and inner stored grain (*σ*_*h-g*_). As illustrated in Figure 1, due to the difference *σ*_*h-s*_ and *σ*_*h-g*_, the silo wall is subjected to a compressive stress as *σ*_*h-s*_ is always large than *σ*_*h-g*_. Considering no-load of grain, i.e., empty-storage condition, the diameter of granary is D (Figure 1a), the wall of a silo with a depth of 30 m may suffer an average horizontal force equal to 30π*Dσ*_*h-s*_ (Figure 1b). Therefore, the wall of underground silo normally bear the combined compressive loading and pore pressure (Figure 1c). Given above reasons, a new structure of wall for UGs which functions well under combined compressive loading and pore pressure is desired and further study is needed.

Lots of scholars have conducted a number of works on leak proof for underground space, such as tunnels and garages, and generally mature measures for waterproof in these projects has been established [15, 16, 17, 18, 19, 20]. Pelz et al. [21] and Su et al. [22] analyzed the advantages and disadvantages of existing underground space waterproofing from the perspective of practice engineering and numerical codes respectively. However, the waterproof method in most civil underground engineering can't be applied in the UGs. The UGs has a zero-leakage requirement of the wall as the stored grain is easily affected by the moisture. Fungi might grow rapidly if the water content of the grain increase to some extent. Previous study has confirmed that Polypropylene (PP) has the advantages of physical properties, corrosion resistance and easy-to-construction, and it also conforms to the standards of plastic products for food contact [23]. Some pilot idea of application of PP in underground granaries has been reported recently by Baryali et al. [24] through a six - month test. They found that the UGs with PP lining is better than the traditional UGs with respect to the temperature control, waterproof, moisture-proof and insect-proof. Yin et al. [25] proved that the bond between threaded polypropylene rods and concrete is excellent through experiments. However, up to now, there is little study about the waterproofing performance of PP for UG was presented.

In this study, a new composite structure, polypropylene - concrete wall (PPCW) for underground silo was proposed. The proposed PPCW can be prefabricated as single slab in the factories and assembled in the field. The concrete guarantees the strength of the silo but with lots of cracks under working condition, and application of PP can prevent water from entering to the silo through the concrete crack. Then, three PPCWs with different size was prepared to test the waterproofing features under the combination of compression and hydropower. According to the experimental analysis, the relationship between the ultimate bearing capacity of the water pressure and the spacing of the studs was given. And a new modified method was proposed to predict the bending moment at mid-span of PPCW by using an adjustment coefficient. This composite structure can work well together, make up for the lack of reinforced concrete structure in waterproof and seepage control, and ensure the waterproof performance of underground granary. In the face of global foodstuff shortages, foodstuff can be stored more safely and environmentally, reducing foodstuff losses and ensuring foodstuff quality.

## 2 Methodology and material

### 2.1 Specimen

**Figure 2:**
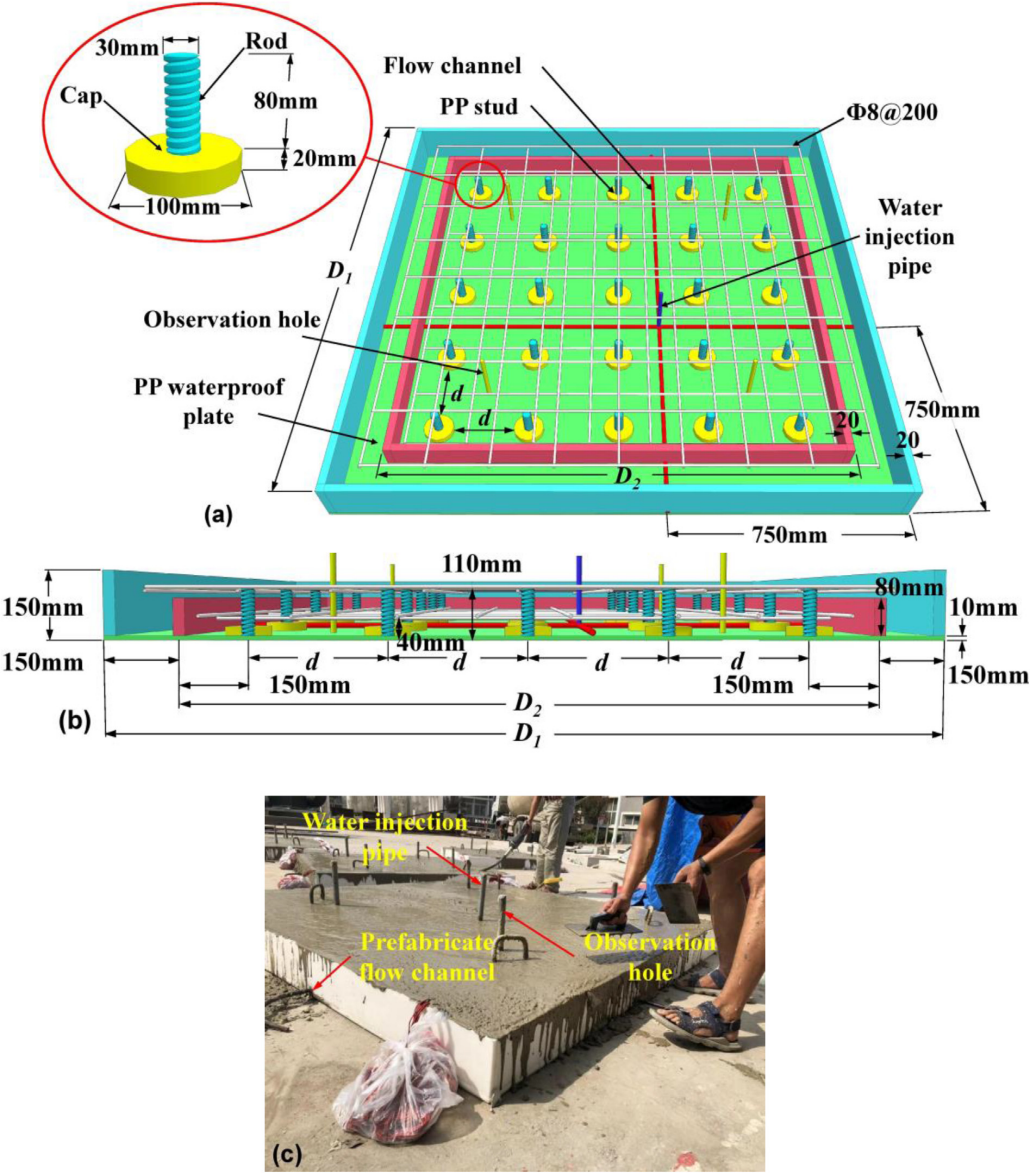
Schematic diagram of the specimen (a) 3D view, (b) elevation and (c) photo.

The proposed polypropylene - concrete wall (PPCW) slab used in the test mainly consists of a PP waterproof board, 25 studs by 5 × 5 on the board (including a cap and rod), two layers of bar-mat reinforcements (Figure 2a). The PP waterproof board has thickness of 10 mm and connected with the stud cap by circular welding around the cap edge. The stud cap is 20 mm in thickness and 100 mm in diameter, and the stud rod is 80 mm in length and 30 mm in diameter with screw threads at 1.5 mm in pitch [26]. The stud is made of the same material as waterproof board, i.e., PP. The bottom and upper layer of bar-mat reinforcements was placed at a height of 40 mm and 110 mm, respectively (Figure 2b). The bar used is HRB400 and has a diameter of 8 mm. The space of bar-mat reinforcements is 200 mm in two-direction.

Around the waterproofing board, a sealing formwork-wall was used. The wall was assembled using 4 PP plates with a thickness of 20 mm and a height of 140 mm. And each plate were welded first and then was further welded with the waterproof board (Figure 2a). Then, by similar method, another inner sealing formwork-wall was used and each side was located 150 mm far from the outer framework-wall. The value of *D*_1_ is 1600, 1800 and 2000 mm for slab A, B and C, respectively. The value of *D*_2_ is 1300, 1500 and 1700 mm for slab A, B and C, respectively. The inner wall was aimed to provide a double waterproofing effect. To simulate the potential crack, two round bars with a diameter of 14 mm were pre-placed on the PP board before pouring concrete, and the surface active agent were brushed onto the surface of the two bars in advance. Then, the steel bars were pulled out just after the initial set to form two flow channels (Figure 2a) and two ends of each channel were plugged by a short round bar and high-strength bonding agent. A steel water injection pipe was embedded at the intersection of the two flow channels. The water injection pipe is to simulate the damage (crack) of the concrete considering the most unfavorable condition and push the water directly into the junction the concrete and PP bolt. Detailed dimensions can be found in Figure 2a and b. Four observation holes made by steel pipe were placed just on the PP board before pouring concrete (Figure 2a and c). The observation holes were used to discharge the residual air in the pipe and ensure a full water filling. On the other hand, the observation holes were used to determine whether there is the water flow passing during testing by monitoring the water pressure in real time using a pressure gauge. Four reinforcement hooks with a diameter of 16 mm were fixed to facilitate lifting and turning during installation (Figure 2c). The total height of the specimens was 150 mm with a 140 mm - thick C40 concrete and a 10 mm – thick PP board.

In this study, 3 specimens. i.e., slab A, B and C were test, and the dimensions of the PP boards are 1600 mm × 1600 mm, 1800 mm × 1800 mm, and 2000 mm × 2000 mm, respectively. The structural setup of the three slabs are generally the same, except for stud spacing (*d*). The value of *d* is 250, 300 and 350 mm for slab A, B and C, respectively. The polypropylene used in this study has a tensile yield stress of 21.5 MPa at yield strain equal to 3.7% according to GB/T 1040.1-2018 [27]. The elasticity modulus of 1430 MPa and Poisson's ratio of 0.48 were finally determined for PP board. At the same time of pouring concrete, six standard cubic blocks with dimensions of 150 mm × 150 mm × 150 mm were cast and cured under the same conditions as the PPCW specimens for 28 days. The compressive strength test was performed, and the average strength of the concrete was 39.14 MPa according to GB 50204–2015 [28].

### 2.2 Monitoring

**Figure 3:**
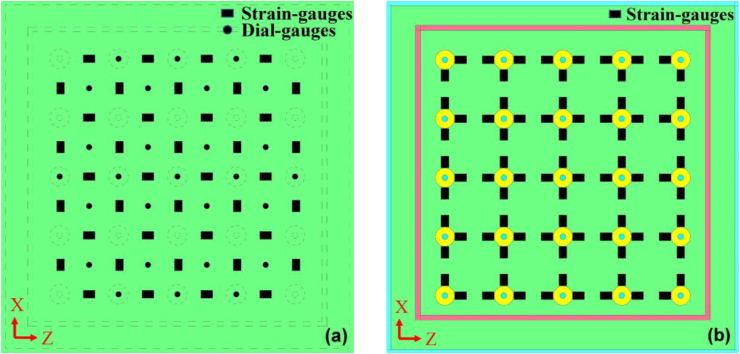
Measuring points on PP board (a) strains on outer surface; (b) strains and displacement on inner surface.

In this research, the strain at the mid-span of two studs on the outer open surface were measured using strain-gauges, see solid rectangular points in Figure 3a. For a strain-gauge, the measured strain direction is along the long-side of the rectangular point. On the same surface, dial-gauges were used to monitor the lateral displacement (*s*_*h*_) whose direction is normal to the PP board surface. The measuring points (i.e., solid dot) are corresponding to some stud centers and all the square centers formed by the 4 near studs, respectively (Figure 3a). Furthermore, strain gauges were pasted near each stud on the inner surface (contacting with concrete) of PP board as shown in Figure 3b. On the concrete surface, three strain gauges were set up to measure the tensile or compressive strain of the concrete.

### 2.3 Test setup and procedure

**Figure 4:**
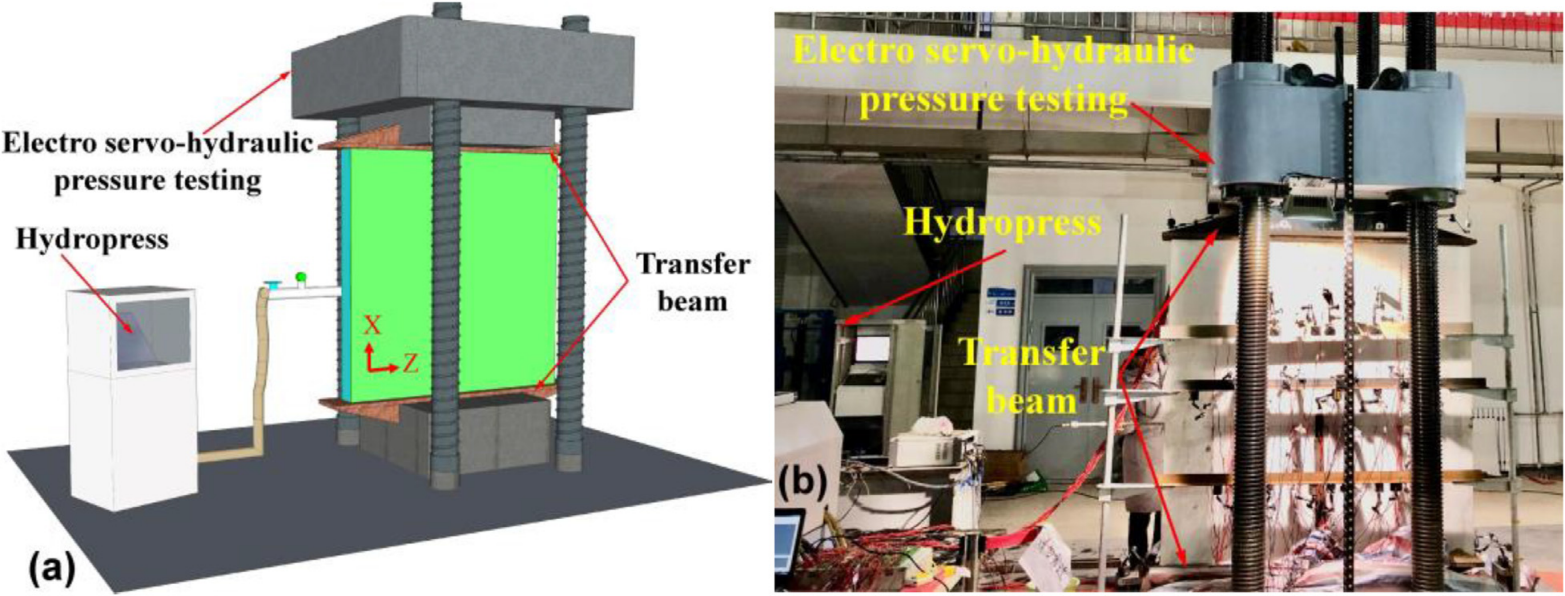
Testing apparatus (a) illustration and (b) photo.

The slab specimens were vertical loaded by the electro servo-hydraulic pressure testing system with a load capacity of 12000 kN (illustrated in Figure 4a). The displacement measurement range of the system is 0–600 mm, accurate to 0.001 mm. As t loading plate of the test machine was smaller than width of the plastic-concrete specimen, two transfer beams were designed and attached to the upper and lower bearing plates of the loading frame, ensuring uniform axial compression loading. Note the vertical loading direction is identical with the X direction as shown in Figure 1c to simulate lateral compression (see Figure 1b). From the water injection hole, pressurized water was pushed into the specimen thorough a hydropress. The pressure range of the hydraulic press is 0–3 MPa, the pressure control accuracy is −1% to 2%, the pressure display is accurate to 0.001 MPa. The applied water pressure can be as a maximum of 3000 kPa. The detailed testing procedure is as follows:

(a) ***Preloading.*** A load of 300 kN was applied gradually with an increment of 50 kN every 3 min. The installation of specimen and working condition of the testing devices and monitoring equipments were checked during stage of preloading.
(b) ***Compressive loading*.** The specimen was vertically loaded to their designed bearing capacity, i.e., 1800, 1900 and 2200 kN for slab A∼C, respectively. The final vertical stress subjected by the specimen is 7.5 MPa, 7.04 MPa and 7.33 MPa for slab A∼C, respectively. The loading for each stage is 200 kN and each stage lasted for 3 min. The hydrotest was performed when the designed bearing capacity was reached.
(c) ***Hydrotest*.** After vertical loading, the hydrotest was performed by application of an incremental water pressure of 10 kPa at an interval of 3 min. The hydro test was determined until failure of the PP board was observed. During the test, the water pressure (*u*) near the water injection hose was measured by a pressure gauge to observe the hydraulic pressure variation. The photo of the test was shown in Figure 4b.

## 3 Results and analysis

### 3.1 Deformation of PP board during compression

**Figure 5:**
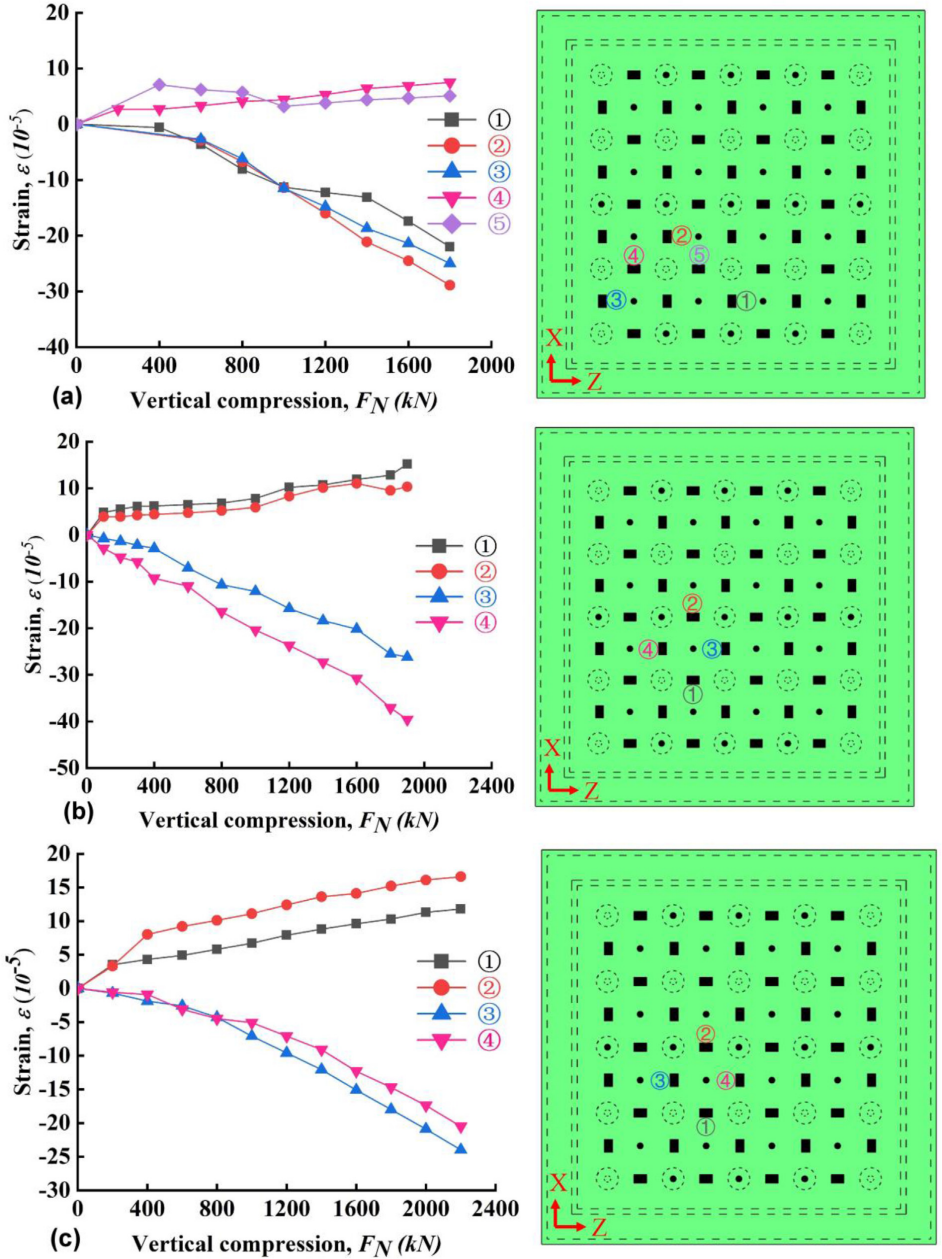
Strain as a function of compressive force at mid-span (a) slab A, (b) slab B and (c) slab C.

**Figure 6:**
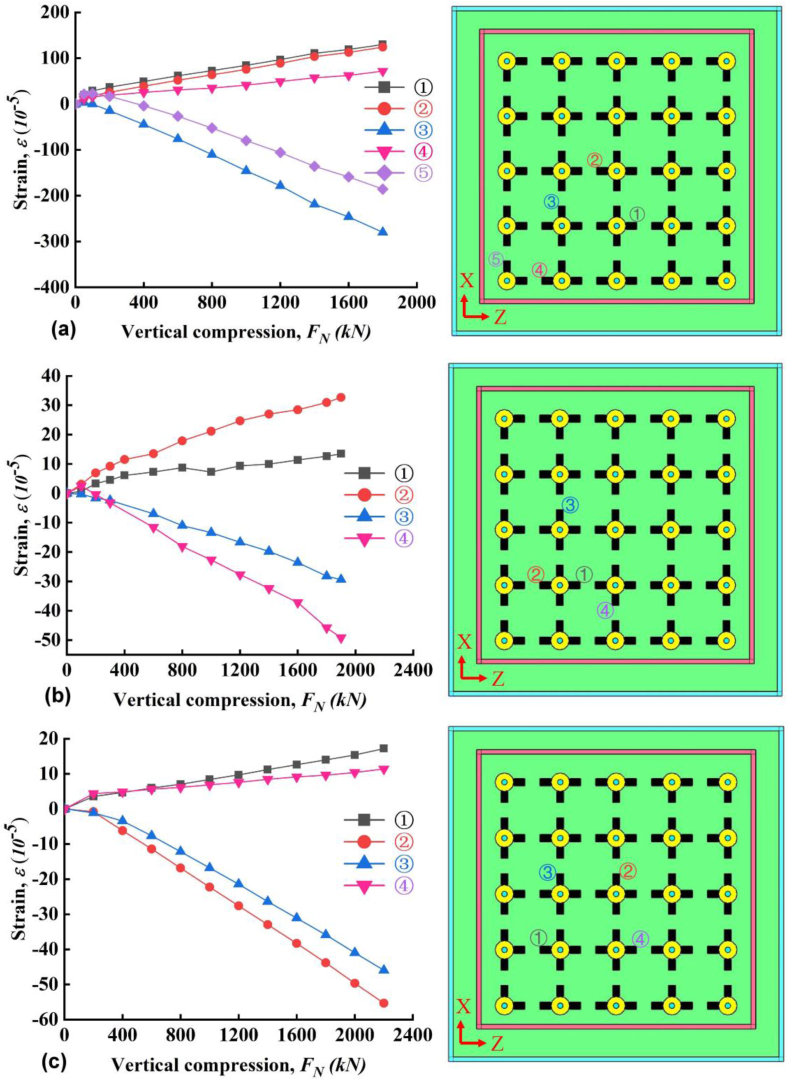
Strain as a function of compressive force near cap (a) slab A, (b) slab B and (c) slab C.

The strains of PP board at the outer surface during vertical compression were shown in Figure 5a–c. Cleary, under vertical compression the PP board presents compressive strain along the vertical direction and tensile strain along the horizontal direction. Furthermore, at different locations, the compressive and tensile strains are identical even with some scatters. The scatters might be due to the production of the board or measurement errors and the differences were acceptable. The strain ∼ stress curves presented a linear relationship in general, the final compressive strain (*ε*_*c*_) is around -3 × 10^−4^ and the tensile strain (*ε*_*t*_) is around 1 × 10^−4^. The ratios of *ε*_*c*_/*ε* are constant generally during the whole vertical loading process. The strains of PP board at the inner surface during vertical compression were shown in Figure 6a–c. The strains at the inner surface followed similar trend to that at the outer surface. The exact values and orders of *ε*_*c*_ and *ε*_*t*_ at both surfaces are very close to each other with a very small magnitude. These results showed that there are no unacceptable warping, buckling and ballooning of the PP board during loading. The good interaction working performance between the PP board and concrete can be indirectly indicated by the above results.

**Figure 7:**
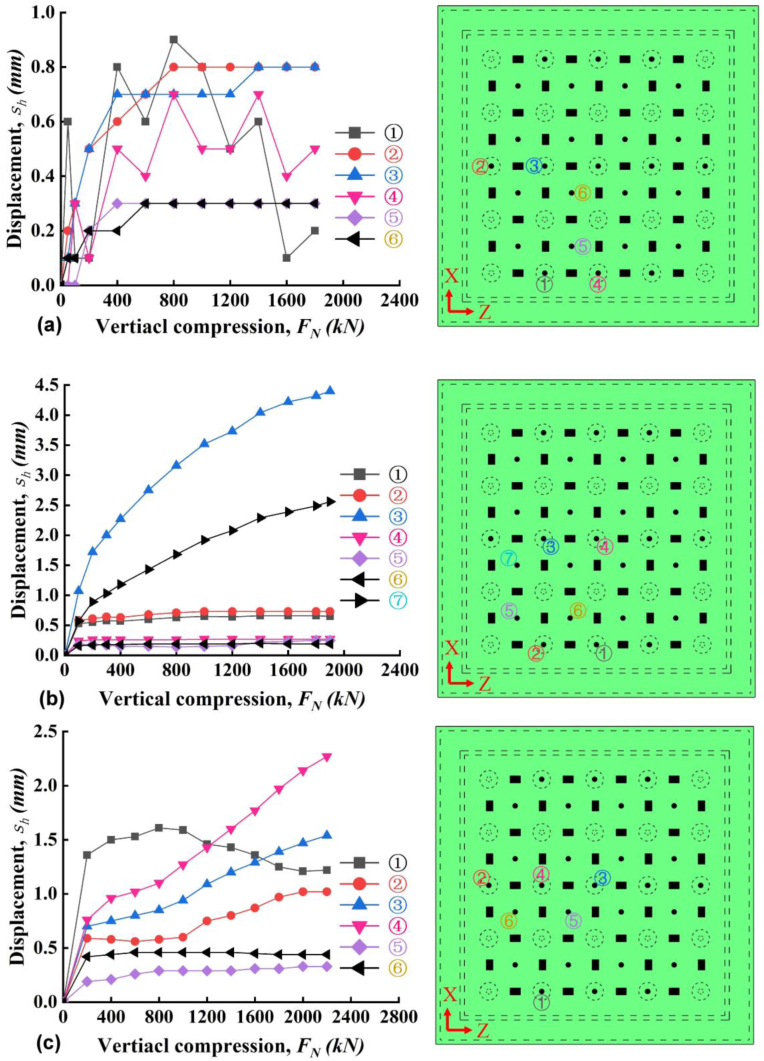
Lateral displacement as a function of compressive force (a) slab A, (b) slab B and (c) slab C.

The lateral displacements (*s*_*h*_) obtained from randomly selected points of the outer surface were plotted in Figure 7. Note that the instrument in slab A seems not stable and the data in Figure 7a fluctuated up and down. The reason is not clear and possibly the instrument or data-logger in slab A was disturbed by an unknown factor. This issue was not existing in B and C, as shown in Figure 7b and c. Anyway, the trend and range of the values are reasonable and the errors were neglected in this study. For slab A, B and C, the maximum *S*_*h*_ is 0.75, 5 and 1.5 mm during vertical compression test, respectively. Note that at the final loading, the applied stress from the servo-hydraulic pressure testing system is close to each other, equal to 7.5, 7.04 and 7.33 MPa for slab A, B and C, respectively. The abnormal maximum *S*_*h*_ at ③ and ⑦ for slab B in Figure 7b indicates that there might be some inner/undetected faults of slab B. These faults was possibly induced by the poor welding quality. And these faults likely affected the performance of water resistance during later hydrotest.

### 3.2 Performance under hydraulic loading

#### 3.2.1 Water pressure variation

**Figure 8:**
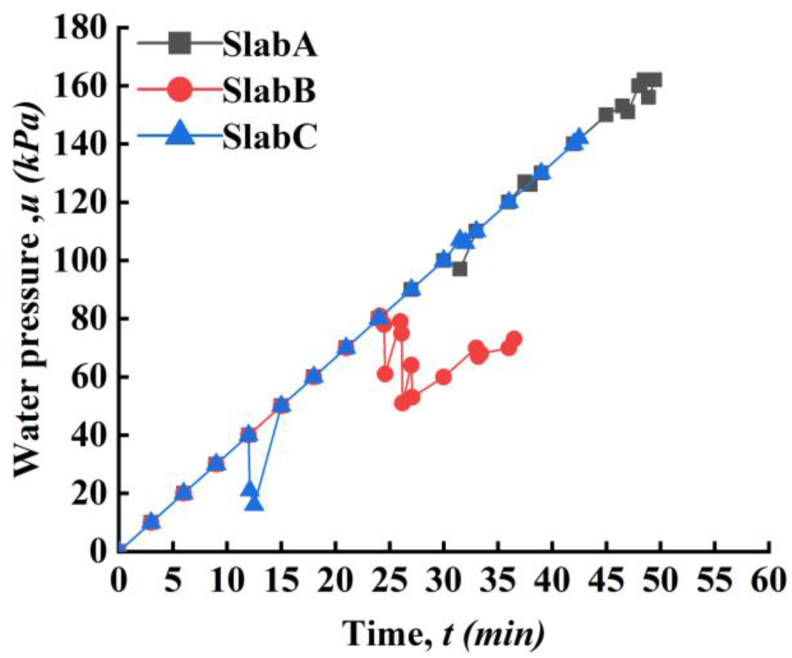
Applied water pressure with time.

**Figure 9:**
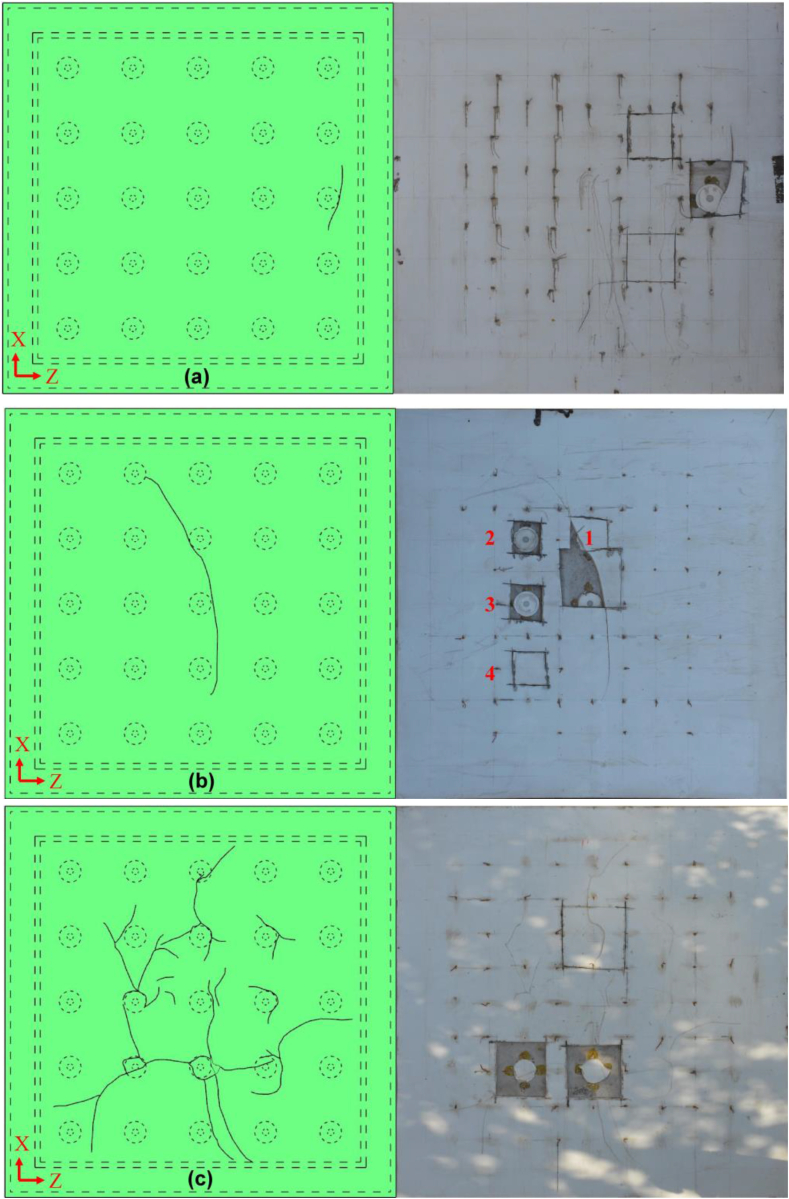
Illustration and photo of crack profile of (a) slab A, (b) slab B and (c) slab C.

The measured water pressure near the injection hole was shown in Figure 8 for all three slabs. For slab A, the water pressure continuously increased with time without any change until the 30th min. There is a slight decline at 30th min corresponding to 100 kPa concurrent with a splintering sound. Then, the water pressure very soon recovered to the loading line, the maximum water pressure (*u*_*max*_) beared by the slab A is 162 kPa. The applied water pressure can't be maintained after 162 kPa and a 15 cm - long crack was found (Figure 9a). Then the water flow out from the crack and the hydrotest was terminated for Slab A. The water pressure for slab B can be maintained (i.e., *u*_*max*_) exactly as applied route until 24th min at 80 kPa. As mentioned above, the slab B may have some inner faults or breakage during vertical compression possibly resulted from poor welding quality. After 24th min, a long crack of PP board (Figure 9b) existed and the water pressure lost its stability thereafter even with continuous pressure supply. After several up-and-downs of water pressure, finally pressurized water flowed out from the crack resulting the failure of slab B. This the reason why slab B has the smallest value of maximum water resistance capacity. For slab C, it was observed that a sharp decline of water pressure at 13th min. The sharp decline might be induced by the bulging of the PP board and followed by the water pressure decrease. However, the PP board in slab C didn't failed at this stage with occurrence of crack. Then, after more water supplied to the enlarged PP-concrete gap, the water pressure very soon recovered to the loading line. The final *u*_*max*_ of slab C is 140 kPa and slab C was failed by multi-cracks appearing almost at same time (Figure 9c).

#### 3.2.2 Profile of cracks and maximum water pressure

The specimen was cut and checked after the test so as to further investigate the local failure of different slabs. The profile and location of the crack was illustrated in Figure 9a. The crack near a stud has a length of approximate to 15 cm. Thus, the authors cut off a square checking-window at the crack from the PP board. After cutting, a fragment of PP board can be easily taken out for the specimen. The connection between the PP board and stud cap (call PP-cap connection) was totally destroyed (Figure 9a) by the pressurized water. Then, the applied pressurized water was fully filling the gap between the PP board and the stud cap. This induced a higher lateral stress at the corresponding area of the gap and finally resulted in the failure of the PP board, i.e., existence of crack. However, another two checking-windows were cut and found there is not fracture of the PP-cap connection.

PP board of slab B also has a crack, while the crack is much longer than that of A. The length of the crack is 4 times of the crack of slab A. Similar to A, we cut the specimen and observed the PP-cap connection at point 1. And a fragment was easily taken off and the connection was totally failed. Furthermore, we cut another three square checking-windows at point 2, 3 and 4. It was surprisingly found that at 2 and 3 the PP-cap connections were also damaged completely. Thus, it was concluded that massive PP-cap connections have already experienced fractures before the PP board's failure due to pressurized water flowing out through the crack.

Different form A and B, slab C has multi-cracks rather than a single crack. The length of cracks is varied and the locations is distributed everywhere in slab C. Three check-windows were cut off to investigate the conditions of PP-cap connection in slab C. The crack is unique and no fracture of PP-cap connection was found. The crack in slab C developed around the stud cap indicating the good performance of the circular welding.

From above results, it was interestingly found that (1) the existence of crack mainly due to fracture of PP-cap connection and yields a decline of water pressure at a moment, and (2) the maximum water resistance, i.e., *u*_*max*_, is linked with the length rather than the numbers of the crack. For slab B where crack with a maximum length, the *u*_*max*_ is the only 80 kPa much smaller than *u*_*max*_ in A and C. If carefully checking, two cracks in C has a longer length than the crack in A, thus the *u*_*max*_ of A is higher than C. It is easy to image that the pressurized water would like to flow out from the weakest point – longest crack and resulted in the failure of waterproofing system. In addition, comparing A and C, slab A has a better waterproofing effect than C. Small stud spacing may enhance the waterproofing of PPCW. Last, from all the three tests, no breakage between stud and concrete was found. Thus, the ultimate performance of PPCW was controlled by welding and strength of PP board.

#### 3.2.3 Lateral displacement

**Figure 10:**
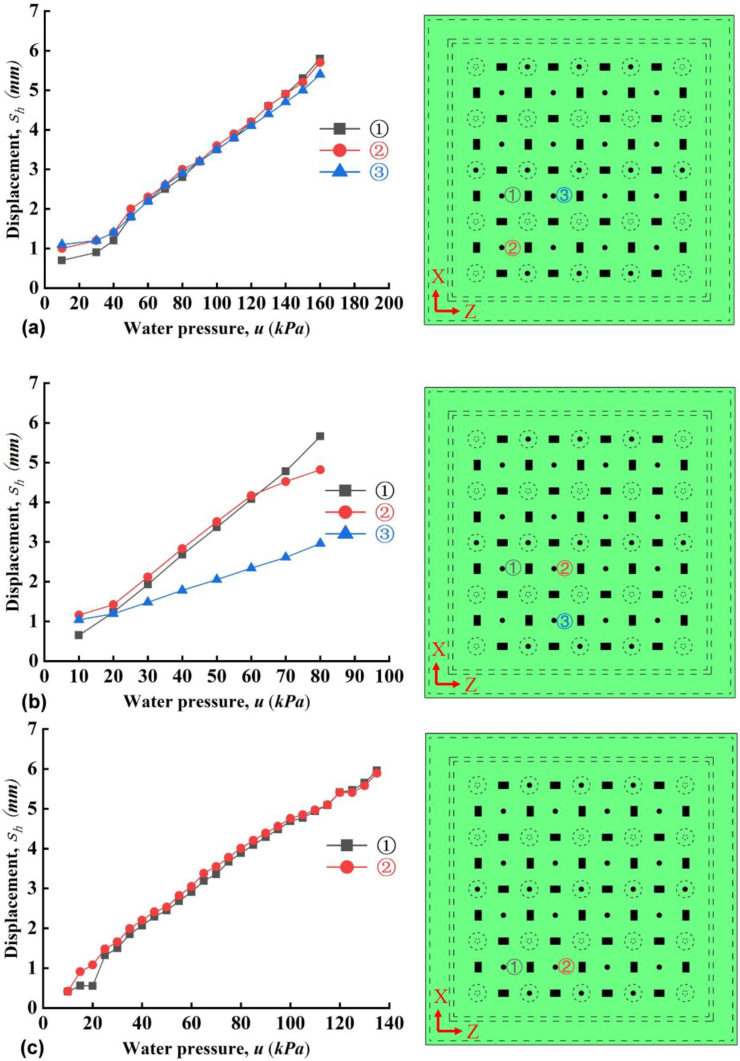
Lateral displacement with water pressure (a) slab A, (b) slab B and (c) slab C.

During hydrotest, the lateral displacements were measured at various points, e.g., at the stud or between the stud. As the stud was embedded into the concrete and presented little displacement, we randomly selected 2–3 points to investigate lateral displacement. First, clearly the displacements increased linearly with the applied water pressure. Furthermore, we can see that the at the failure stage in Figure 10a–c, the maximum lateral displacement of all three is identical, i.e., around 6 mm. However, the slope of the *s*_*h*_ *∼ u* curve is different with different slabs. Without considering slab B, the slope in slab A is smaller than that in slab C. The stud spacing of A is smaller than C, thus the deformation of PP board of A has smaller displacement under same magnitude of applied water pressure, as shown in Figure 10a and c.

## 4 Analysis of stress on PP board

### 4.1 Simplified analysis unit

**Figure 11:**
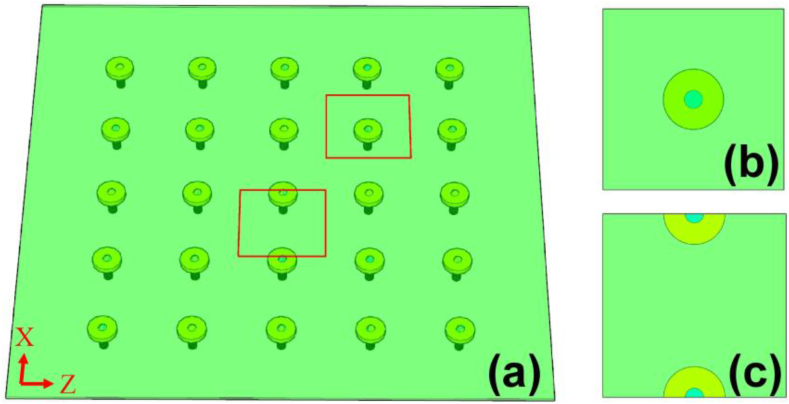
Analysis model (a) plastic components, (b) square unit and (c) span unit.

In this section, we adopted a column-cap-supported plate model to analysis the stress and bending on the PP board. The plastic components of the PPCW are made up of PP board, cap and stud (Figure 11a). In order to analyze the average stress taken by single circular-welding of a stud, a square unit was assumed as shown in Figure 11b. Furthermore, the PP board was subjected to significant bending under water pressure. An analysis unit – a span unit (Figure 11c) between two studs was adopted to investigate the bending moment.

### 4.2 Lateral stress

Assume that the PP board and concrete are working individually under water pressure and the water pressure can be fully applied on the PP board during hydrotest. For a square unit (Figure 11b), the acting area of the pressurized water is equal to 54646, 82146 and 114646 mm^2^ for A, B and C if no crack, respectively. Thus, every welding of a cap in A was subjected to a maximum water pressure of 8.85 kN under the applied maximum water pressure of 162 kPa, and this value is 6.57 kN and 15.47 kN for slab B and C under 80 and 135 kPa of water pressure, respectively. The PP-cap connection of slab B was subjected to the lowest average force. Considering above results of crack features, it is again to known that the welding strength of slab B didn't reach the design capacity and the welding quality was not well controlled. Slab C maybe presented the best welding performance, the PP board was broken under 15.47 kN without fracture of PP-cap connection.

### 4.3 Bending moment at mid-span

**Figure 12:**
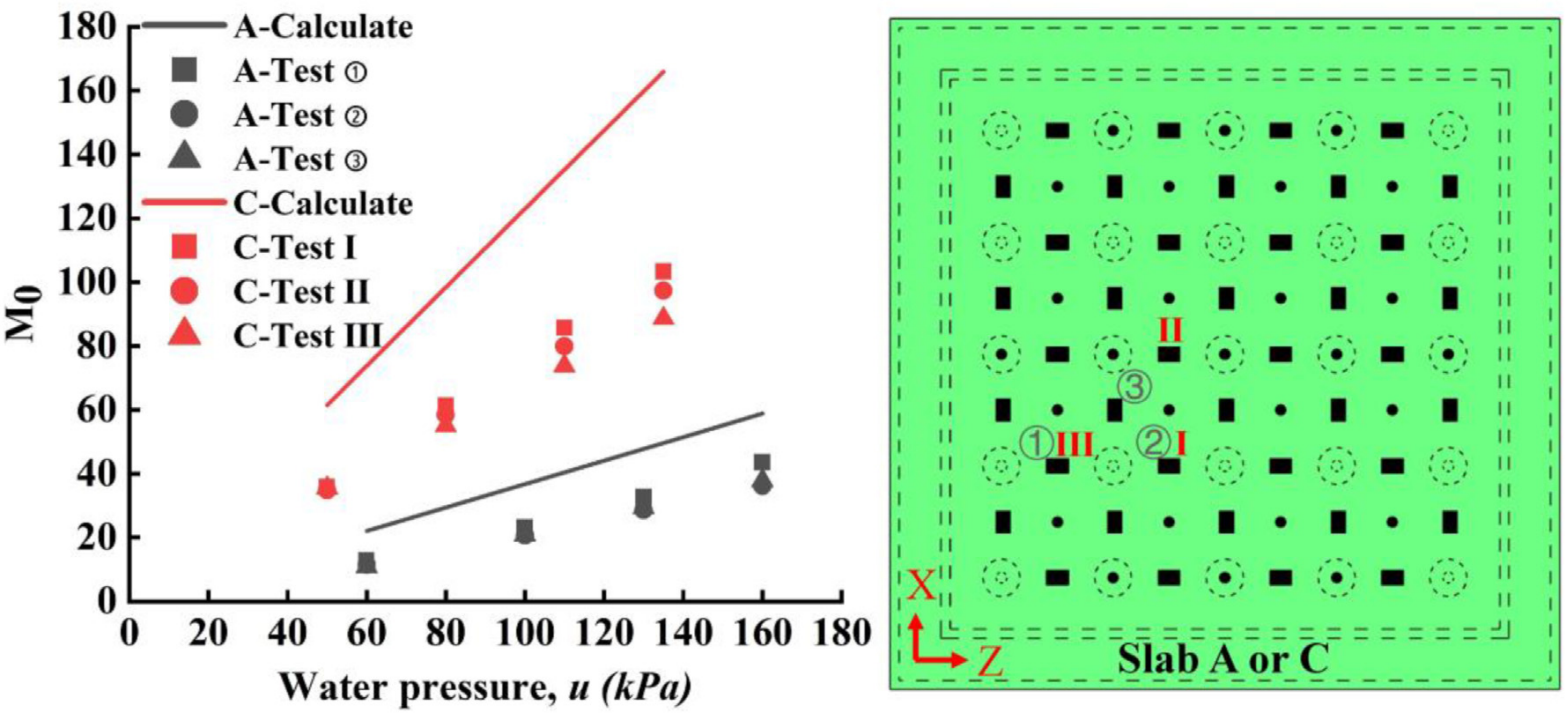
Bending moment from empirical coefficient method and test measurement.

According to empirical coefficient method [29], the total static bending moment, *M*_0_, of the span-unit (Figure 10c) under the vertical uniform load can be calculated by the following formula:

(1) $$M_{0y}=\frac{1}{8}{pl}_{x}{\left(l_{z}-\frac{2}{3}c\right)}^{2}$$

(2) $$M_{0y}=\frac{1}{8}{pl}_{y}z{\left(l_{x}-\frac{2}{3}c\right)}^{2}$$

where *l*_*x*_, *l*_*z*_ is the distance between the center of two adjacent stud in *x* and *z* direction. In this study, *l*_*x*_ = *l*_*z*_ = 250, 300 and 350 mm for the three specimens, respectively. *p* is the vertical uniform load which is regarded as the water pressure responding to different stress conditions. c is the effective width of the stud cap which can be estimated as the diameter of the cap which is the *c* = 100 mm. The solution of total static bending moment in Eq. (1(1) and (2)(2) was also called direct design method of flat slab under uniform distributed stress. With *l*_*x*_ equal to *l*_*z*_ the bending moments in the X and Z directions are equal, thus here we only analyze the bending moment in one direction. Based on the direct design method for column-supported plate, the magnitude of the positive bending moment at the mid-span is 0.35$\left|M_{0}\right|$. In this study, the value of *M*_0_ for slab A and C with applied water pressures is shown in Fig. 12. Note that as slab B has the problem of welding, thus the slab B was not included in the analytical investigation.

For each slab, the value of bending moment at the mid-span can also be calculated according to Eq. (3) from measured strains. Eq. (3) is

(3) $$M=\frac{bE\varepsilon h^{2}}{6}$$

where *b* is the width of analysis unit, *h* is the length of analysis unit, *E* is the elastic modulus of PP, *ε* is the strain of mid-span and was assumed to be the representative strain of the span unit. The bending moment at the mid-span was calculated under water pressure of 60, 100, 130 and 160 kPa for slab A and 50, 80, 110 and 135 kPa for slab C, respectively. The calculated bending moment from tested results were also plotted along water pressure in Figure 12. Clearly, the calculated bending moments from measured strains and direct design method don't match with each other. This is due to the natural different between the concrete column-support flat slab and the stud-cap-PP board. First, the connection of column and flat slab are cast in-situ, while the PP-cap connection are circular welds around the cap. The strength and deformation properties of these two joints are totally different. Second, the material of concrete column-support flat slab and the stud-cap-PP board are different. The material properties differentiates from each other completely.

**Figure 13:**
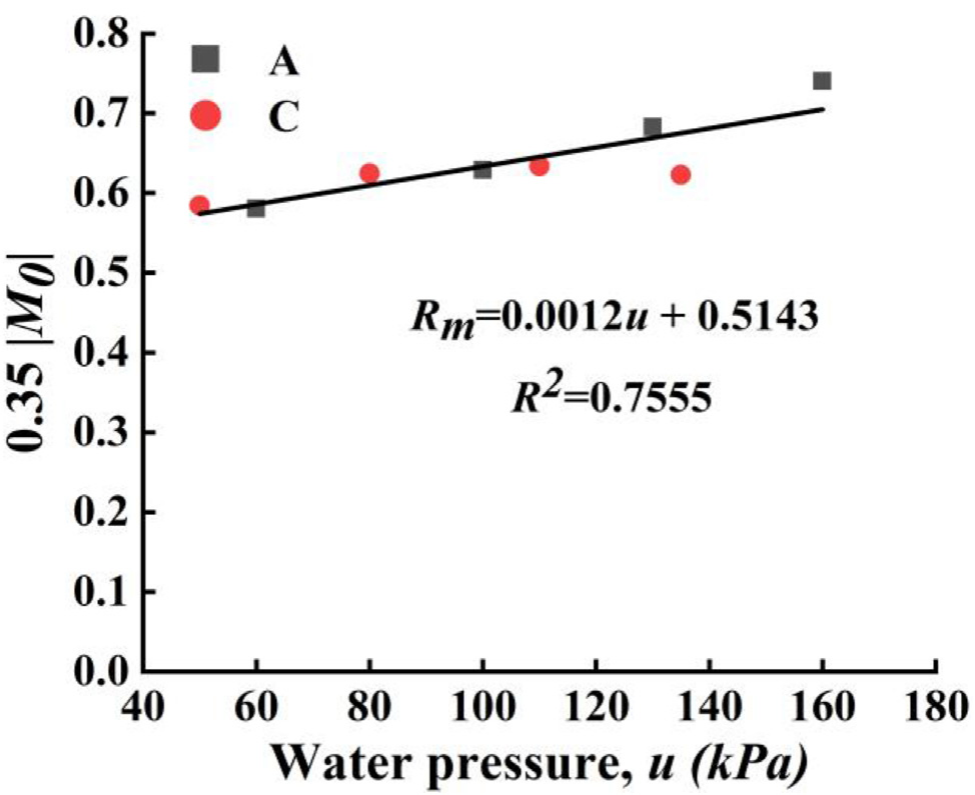
The fitting line of *R*_*m*_ with water pressure.

The ratios (*R*_*m*_) of bending moments from measured strains to that from empirical coefficient method were calculated and plotted in Figure 13. First, it was interestingly found that the value of *R*_*m*_ is located in a small range from 0.55 to 0.75 with an average of 0.64. That is to say that if empirical coefficient method was used to estimate the bending moment of mid-span, adopting a *R*_*m*_ = 0.64 could control the relative errors under 15.6%. Furthermore, the *R*_*m*_ is increasing with applied water pressure. From the fitting line, the value of *R*_*m*_ as a function of water pressure, *u* can be expressed as Eq. (4):

(4) $$R_{m}=0.0012u+0.5143$$

And the bending moment at mid-span of the PP board in the proposed PPCW of this study can be estimated as Eq. (5):

(5) $$M_{\text{pp}}=\left(0.00007u+0.03\right)bE\varepsilon h^{2}$$

where the subscript (pp) refers the PP board. This empirical equation can provide a preliminary evaluation (or trail and error) of internal force of PP board before design or numerical calculation. And it could also be used as simple method to estimate the bending moment of PP board under the circumstance of no-available finite element tools.

## 5 Numerical codes analysis

### 5.1 Constitutive model and established of parts

**Figure 14:**
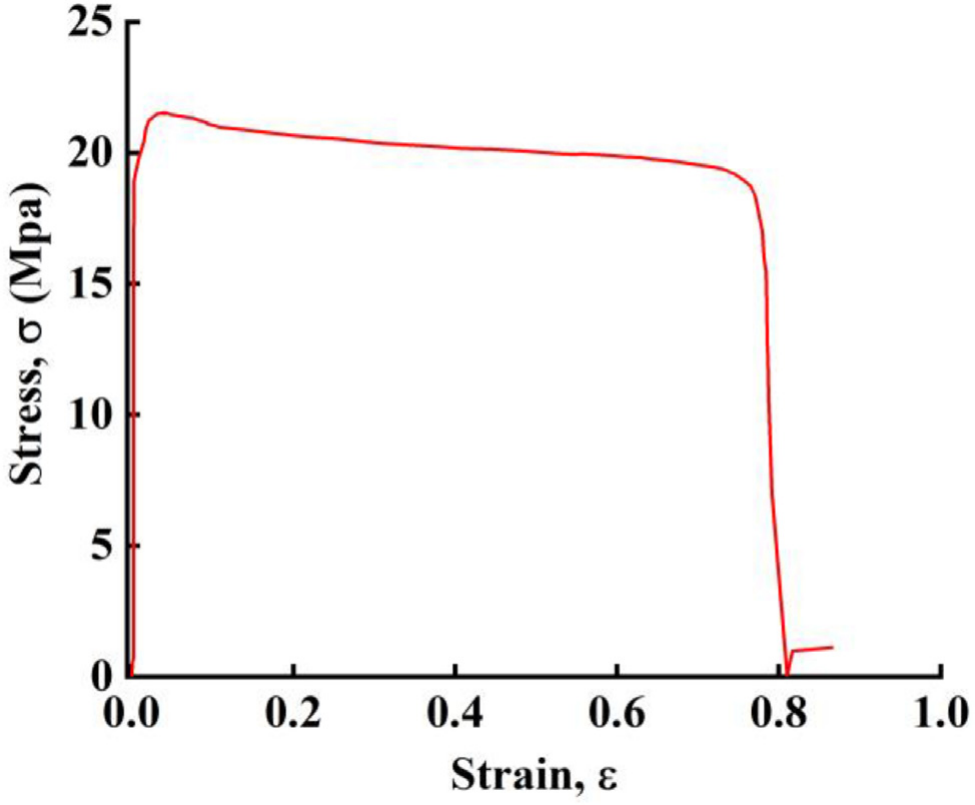
The constitutive model of PP board.

The finite element software, ABAQUS [30], was used for numerical simulation. In this study, the constitutive model of concrete adopted the Concrete Damage Plasticity (CDP) mode, which is suggested by Lubliner et al. [31] and Lee et al. [32]. The tensile and compressive damage of the concrete in this model are described by using different damage factors. The CDP yield a better convergence of calculation in simulating the tension and compression of the concrete. The constitutive model of the PP board, as shown in Figure 14, was obtained from the material tests and used in this simulation.

**Figure 15:**
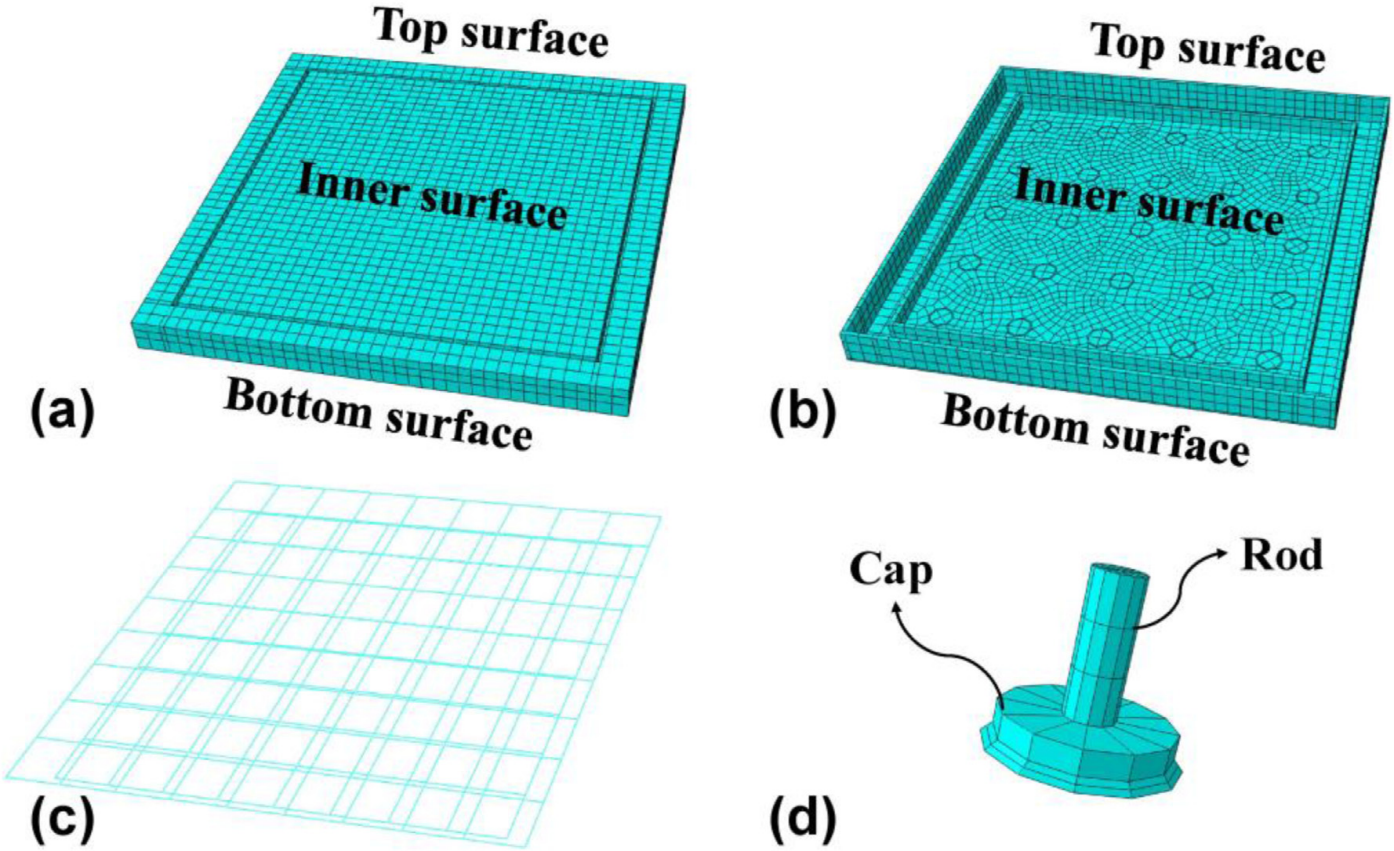
The model of parts (a) concrete, (b) PP, (c) steel-bar mesh, (d) stud.

Based on the experimental result, numerical model for Slab C is established by ABAQUS first and compared with tests to verify its effectiveness. The dimensions of the concrete, stud (cap and rod), PP board and steel-bar mesh are set up respectively according to the test. Note the flow channels, observation holes and water injection holes are not modeled in the study. Instead, we applied a uniform water pressure on the inner surface of the PP board. For reduce the calculation time, the seeds of the mesh is 50 mm for concrete (Figure 15a), PP board (Figure 15b) and steel-bar mesh (Figure 15c), and the seeds of mesh is 30mm for studs (Figure 15d).

**Figure 16:**
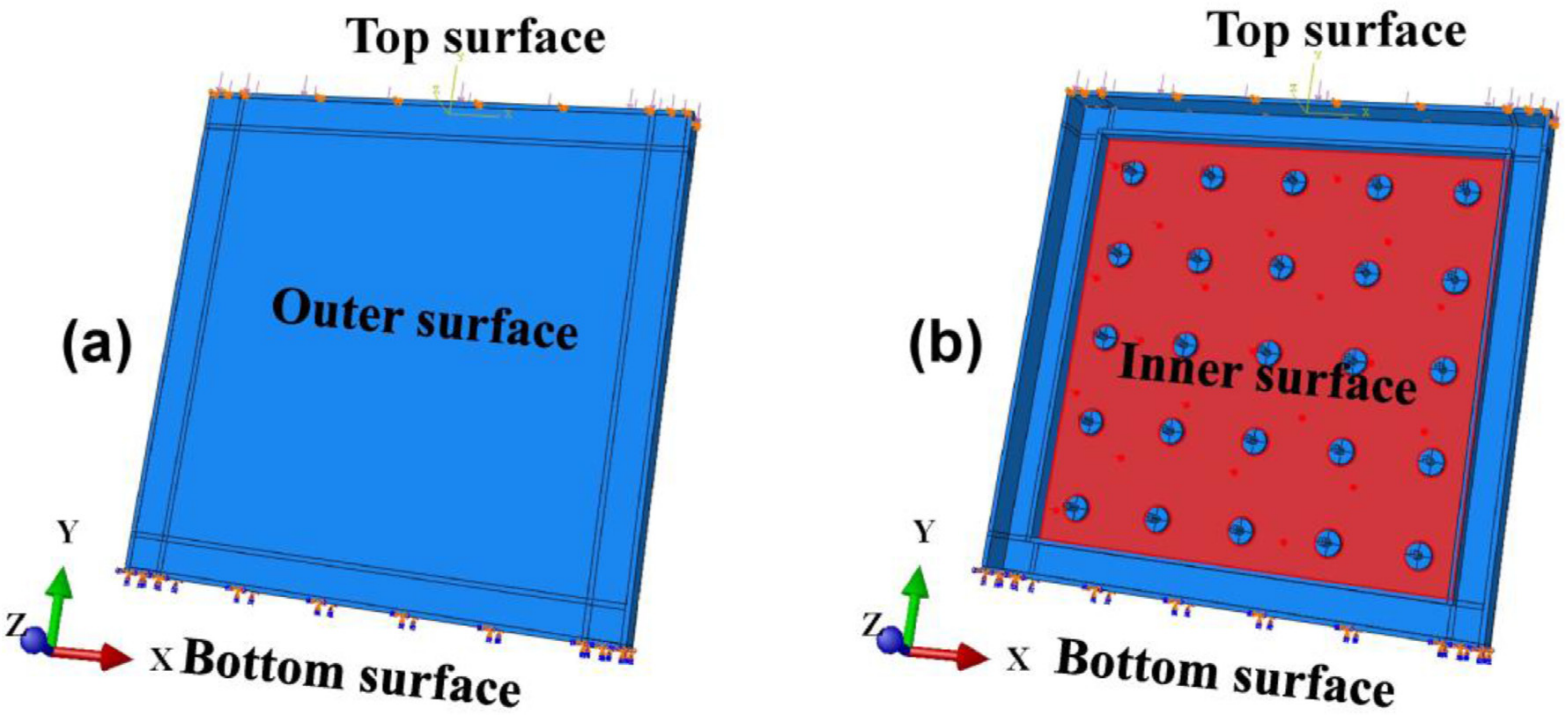
The setting of load and boundary condition, (a) Step-1, (b) Step-2.

The contacting interface of (1) the PP board and the concrete, (2) the stud cap and concrete, (3) steel-bar mesh and the concrete, and (4) stud cap and PP board (i.e., connection) were need to be considered in the ABAQUS. The concrete is in contact with the inner side of PP board, the property of the interaction includes tangential behavior and normal behavior. Tangential behavior is ‘Penalty’ and normal behavior is ‘Hard contact’. The steel-bar mesh and stud (cap and rod) are ‘Embedded’ in the concrete. And the weld of stud (i.e., PP board-cap connection) are ‘Tie’. The simulation has two steps, in step-1 (Figure 16a), the bottom surface of model is ‘Encastre’, and the pressure along the negative of the Y-axis is applied to the top surface of model. The loading applied is completely identical with the experiments. In step-2 (Figure 16b), the vertical load was hold with no increment and applied the uniform pressure on the contacting surface between the PP board and the concreted. The load and boundary condition are shown in Figure 16.

### 5.2 Comparison with test results

**Figure 17:**
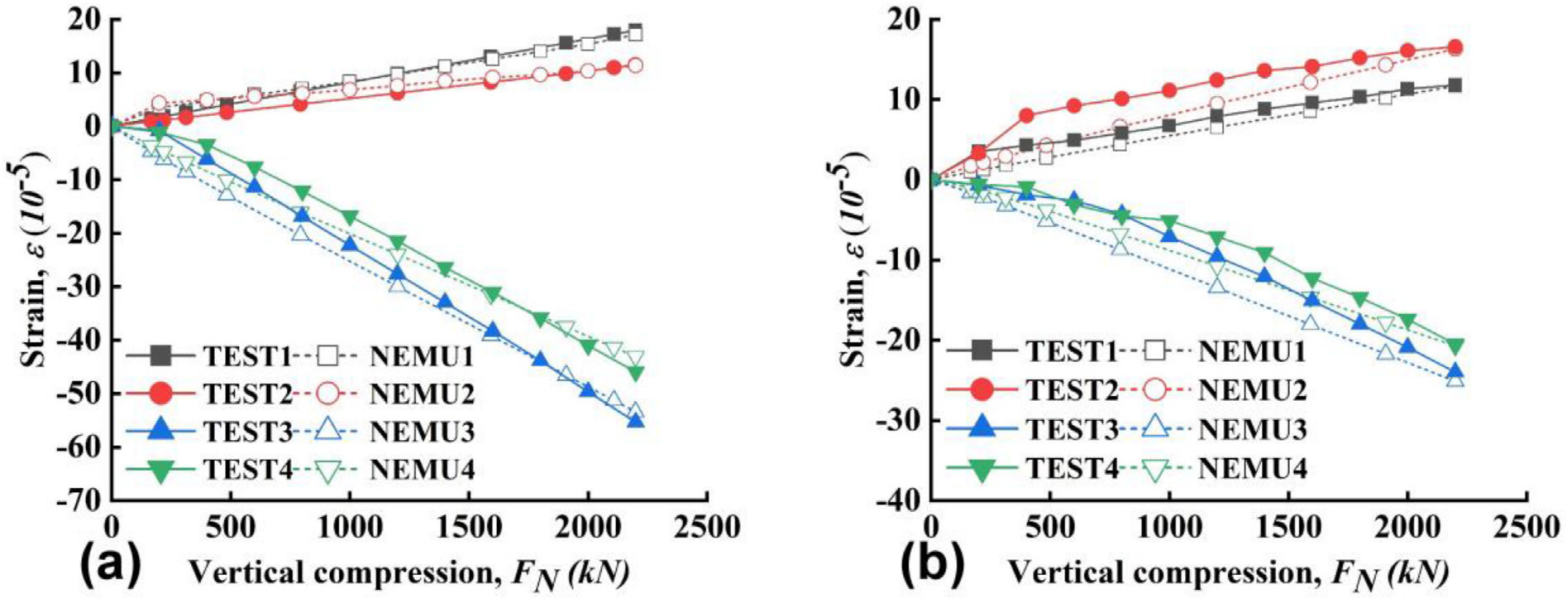
The strain of comparison (a) internal side of PP, (b) external side of PP.

**Figure 18:**
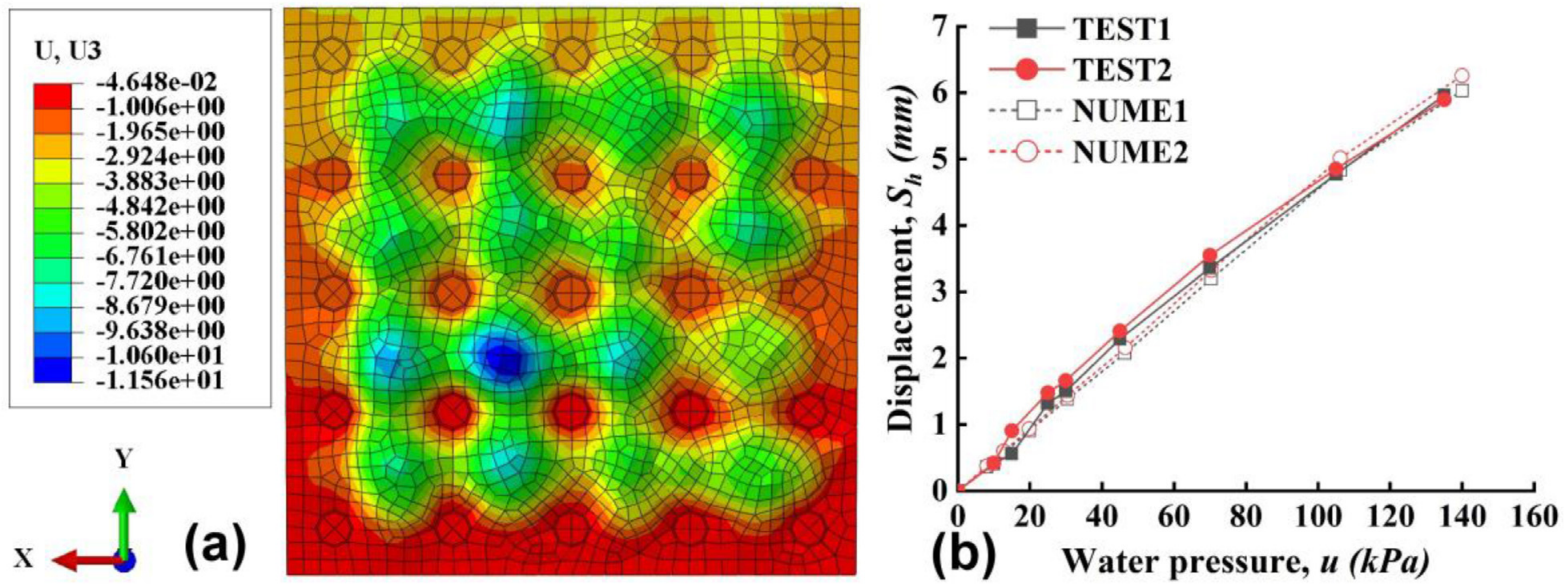
The displacement of hydrotest (a) displacement cloud, (b) comparison vertical loading stage.

First, the compressive and tensile strain of PP board during vertical loading from simulation and tests were compared and the results are shown in Figure 17a and b. The strain from the tests is the same as that in Figures 5c and 6c. Even with little difference, the simulation results have very good consistency with the test results. The difference of between simulated results and tested results was possibly induced by the flaw of the tested slab during manufacture. Second, during the hydrotest, the displacement cloud from simulation and the lateral displacement of the slab C in the mid-span from test and simulation are compared in Figure 18a, b. The tested data is from Figure 10c. We can see that the lateral displacements from the simulation under water pressure agree with the tested ones perfectly. Thus, the method of water pressure application used in this study is reasonable and could fairly simulate the test. As a conclusion from Figures 17 and 18, the model established can reasonably simulate the experimental displacement and can be used in later parameter study.

### 5.3 Parameters study

In this section, we selected to study the effect of the spacing of stud (*d*_*s*_), diameter of cap (*d*_*c*_) and the rod (*d*_*r*_) on the maximum displacement (*S*_*h-max*_). The parameter adopted in finite element analysis is shown in Table 1 and the model established in the above section was used.

**Table 1:** Parameter analysis model.

Number	Spacing of stud d_s_ (mm)	Diameter of cap d_c_ (mm)	Diameter of rod d_r_ (mm)	Max-displacement S_h-max_ (mm)
1	200	100	30	0.05208
2	250			1.24888
3	300			4.55709
4	350			10.62396
5	400			13.12593
6	450			30.92418
7	500			43.12128
8	350	40	30	62.94376
9		60		38.42214
10		80		15.47681
11		100		10.62396
12		120		4.65099
13		140		1.75903
14		160		1.85295
15		180		1.84173
16	350	100	10	10.62417
17			20	10.62385
18			30	10.62385
19			40	10.62379
20			50	10.62379
21			60	10.62378
22			70	10.62386

**Figure 19:**
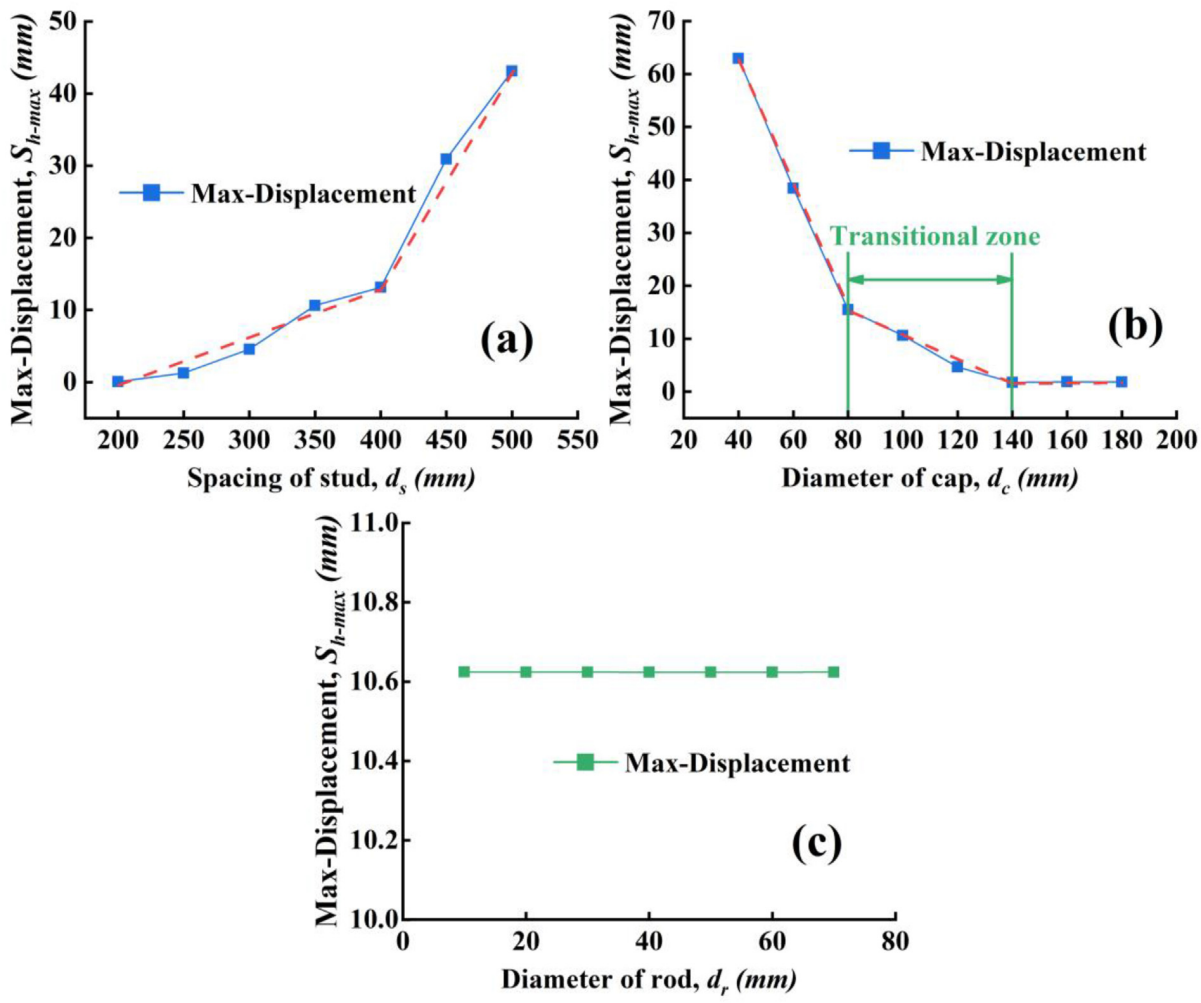
The influence of the parameters on the Max-Displacement. (a) Spacing of stud, (b) diameter of cap, (c) diameter of rod.

The simulated results of *S*_*h-max*_ considering various *d*_*s*_, *d*_*c*_ and *d*_*r*_ are shown in Figure 19a–c, respectively. The maximum lateral displacement of the model decreases with the stud spacing. When the spacing exceeds 400 mm, the maximum lateral displacement of the model increases sharply. Thus, with fixed *d*_*c*_ and *d*_*r*_, the *d*_*s*_ smaller than 400 mm can control the *S*_*h-max*_ under 13 mm. And beyond 400 mm, the rapid increase of the *S*_*h-max*_ may yield significantly negative effect on the PPCW behavior under combined vertical compression and lateral water pressure. As for the effect of the *d*_*c*_, there are two turning points, (1) 80 mm and (2) 140 mm, as shown in Figure 19b. When the *d*_*c*_ is smaller than 80 mm, the maximum lateral displacement of the model increases sharply, and when the *d*_*c*_ exceeds 140mm, increasing the diameter has no effect on the maximum lateral displacement of the model. From Figure 19c, the *d*_*r*_ is not sensitive to the maximum lateral displacement of the model as the *S*_*h-max*_ has no change with *d*_*r*_. Note this result is only true under the condition of this study with the selected parameters in Table 1. In engineering practice, on the premise of satisfying the tensile strength of the rod, it is suggested to choose the *d*_*s*_ below 400mm and the *d*_*c*_ above 80 mm and within 140mm to ensure a basic economy design.

## 6 Discussion and limitation

The water injection hole was set up to consider the most unfavorable condition. But in the real cases, the underground silo may not be the same as the set at the test. In addition, the failure of plate B is due to the poor weld quality and how to guarantee weld quality is an urgent issue in the future. The wall of underground silo is subjected to four types of load respectively due to soil, underground water, foodstuff and the vertical load due to gravity in service. In the future study, performance of PPCW under multi conditions should be investigated.

## 7 Conclusions

In this study, the performance of a new proposed composite structure, polypropylene - concrete wall (PPCW) for underground silo was investigated experimentally. And a new modified method was proposed to predict the bending moment at mid-span. According to the experimental and analytical results, the following conclusions can be drawn.

(1) The compressive strain and the tensile strain at both surfaces of polypropylene (PP) board are very close to each other with a very small magnitude. The PP board and concrete have very good performance of interaction working under compression.
(2) Cracks were yielded under pressurized water and finally resulted the failure of the PPCW. The longest crack dominated the maximum resistance of water pressure and longer the crack is and less the maximum resistance of water pressure is. However, the existence and failure of PP board were significantly affected by the welding quality. And poor welding quality will cause fracture of PP-cap connection and decrease the waterproofing capacity. In general, small stud spacing may enhance the waterproofing of PPCW. The deformation of PP board of slab A with small stud spacing has smaller displacement than C under same magnitude of applied water pressure.
(3) Based on the empirical coefficient method of concrete flat-slab and tested results, a new modified method was proposed to predict the bending moment at mid-span of PPCW by using an adjustment coefficient (ratio of bending moment from test to the analytical solution, *R*_*m*_). Considering this case only, it was found that *R*_*m*_ is from 0.55 – 0.75 with an average of 0.64. And adopting a *R*_*m*_ = 0.64 could control the relative errors under 15.6%.

## Declarations

### Author contribution statement

Hao Zhang: Conceived and designed the experiments; Analyzed and interpreted the data; Contributed reagents, materials, analysis tools or data.

Hongkai Wang: Performed the experiments; Analyzed and interpreted the data; Wrote the paper.

Yang Zhou: Analyzed and interpreted the data; Contributed reagents, materials, analysis tools or data; Wrote the paper.

Zhe Chang: Performed the experiments.

### Funding statement

The work was supported by the National Ministry of Science and Technology 2014 special project for Food Public Welfare Industry (201413007), Scientific and Technological Breakthrough of Henan Province (202102110122), Backbone Training Program of Young Researcher in 10.13039/501100003489Henan University of Technology, Innovative Funds Plan of 10.13039/501100003489Henan University of Technology (2020ZKCJ05) and Special Fund Project from 10.13039/501100003489Henan University of Technology (2016QNJH23).

### Data availability statement

Data will be made available on request.

### Declaration of interest's statement

The authors declare no conflict of interest.

### Additional information

No additional information is available for this paper.
